# The Case for Adaptive Neuromodulation to Treat Severe Intractable Mental Disorders

**DOI:** 10.3389/fnins.2019.00152

**Published:** 2019-02-26

**Authors:** Nicole R. Provenza, Evan R. Matteson, Anusha B. Allawala, Adriel Barrios-Anderson, Sameer A. Sheth, Ashwin Viswanathan, Elizabeth McIngvale, Eric A. Storch, Michael J. Frank, Nicole C. R. McLaughlin, Jeffrey F. Cohn, Wayne K. Goodman, David A. Borton

**Affiliations:** ^1^Brown University School of Engineering, Providence, RI, United States; ^2^Charles Stark Draper Laboratory, Cambridge, MA, United States; ^3^Psychiatric Neurosurgery Program at Butler Hospital, The Warren Alpert Medical School of Brown University, Providence, RI, United States; ^4^Department of Neurosurgery, Baylor College of Medicine, Houston, TX, United States; ^5^Menninger Department of Psychiatry and Behavioral Sciences, Baylor College of Medicine, Houston, TX, United States; ^6^Department of Cognitive, Linguistic, and Psychological Sciences, Brown University, Providence, RI, United States; ^7^Department of Psychology, University of Pittsburgh, Pittsburgh, PA, United States; ^8^Carney Institute for Brain Science, Brown University, Providence, RI, United States; ^9^Department of Veterans Affairs, Providence Medical Center, Center for Neurorestoration and Neurotechnology, Providence, RI, United States

**Keywords:** responsive neuromodulation, mental disorders, adaptive deep brain stimulation, obsessive compulsive disorder, biomarkers

## Abstract

Mental disorders are a leading cause of disability worldwide, and available treatments have limited efficacy for severe cases unresponsive to conventional therapies. Neurosurgical interventions, such as lesioning procedures, have shown success in treating refractory cases of mental illness, but may have irreversible side effects. Neuromodulation therapies, specifically Deep Brain Stimulation (DBS), may offer similar therapeutic benefits using a reversible (explantable) and adjustable platform. Early DBS trials have been promising, however, pivotal clinical trials have failed to date. These failures may be attributed to targeting, patient selection, or the “open-loop” nature of DBS, where stimulation parameters are chosen *ad hoc* during infrequent visits to the clinician’s office that take place weeks to months apart. Further, the tonic continuous stimulation fails to address the dynamic nature of mental illness; symptoms often fluctuate over minutes to days. Additionally, stimulation-based interventions can cause undesirable effects if applied when not needed. A responsive, adaptive DBS (aDBS) system may improve efficacy by titrating stimulation parameters in response to neural signatures (i.e., biomarkers) related to symptoms and side effects. Here, we present rationale for the development of a responsive DBS system for treatment of refractory mental illness, detail a strategic approach for identification of electrophysiological and behavioral biomarkers of mental illness, and discuss opportunities for future technological developments that may harness aDBS to deliver improved therapy.

## Introduction

Mental illness is a leading cause of disability and mortality that affects approximately 13–17% of individuals worldwide (Insel, 2009; Whiteford et al., 2013; Steel et al., 2014; Polanczyk et al., 2015; Walker et al., 2015; Vigo et al., 2016). While significant advances have been made over the last few decades in the development of diagnostic categories and treatment for psychiatric illnesses, many individuals fail to respond to first-line pharmaceutical and behavioral therapy (Rush and John Rush, 2007; Shah et al., 2008; Krystal and State, 2014; Widge et al., 2017). Standard treatments of mental illness often lack anatomical and functional specificity, which may be responsible for limited efficacy and significant side effect profiles, and provide limited data concerning pathological circuitry underlying psychiatric disease (Pittenger et al., 2005; Hofmann and Smits, 2008; Insel et al., 2010; Krystal and State, 2014; Locher et al., 2017; Widge et al., 2017). Development of improved therapies will require a better understanding of the pathological neural activity underlying mental illness.

Neurosurgical interventions such as Deep Brain Stimulation (DBS) have proven helpful in uncovering and confirming the underlying neurocircuitry of several common psychiatric illnesses (Greenberg et al., 2003; Rauch et al., 2006; Romanelli et al., 2014; Widge et al., 2017). This is especially true for Obsessive-Compulsive Disorder (OCD), a psychiatric illness marked by recurrent unwanted or distressing thoughts (obsessions) and/or repetitive, ritualistic behaviors (compulsions) that affects 2.3% of the United States population (Rasmussen and Eisen, 1992; Ruscio et al., 2010). Approximately 10–20% of OCD patients have treatment refractory illness. Stereotactic neurosurgical treatment has proven beneficial for severe, chronic, and otherwise intractable OCD (Greenberg et al., 2003; Shah et al., 2008; Romanelli et al., 2014; Widge et al., 2017). DBS efficacy is similar to that of neuroablative procedures in treating OCD (Greenberg et al., 2010; Brown et al., 2016; Rasmussen et al., 2018). In preliminary studies, DBS in the ventral capsule/ventral striatum (VC/VS) has been found to markedly improve OCD symptoms in approximately 46–73% of patients, potentially through disruption of neural activity in pathways connecting subcortical structures to prefrontal cortices (Wichmann and Delong, 2006; Greenberg et al., 2010; Cleary et al., 2015; Pepper et al., 2015; Brown et al., 2016; McLaughlin et al., 2016; Graat et al., 2017). However, the true mechanism by which DBS improves symptoms remains unclear (Nambu and Chiken, 2014; Widge et al., 2017).

Despite success with neurosurgical interventions for treatment-refractory OCD, the efficacy of meaningful reduction in OC symptoms has room for improvement (Brown et al., 2016; Widge et al., 2017; Rasmussen et al., 2018). This may be due to multiple factors, including failed circuit targeting, patient heterogeneity, and the “open-loop” nature of the current electrical stimulation paradigm. Currently, stimulation parameters are adjusted only on infrequent visits to the clinicians office, and untouched for weeks or months (Wichmann and Delong, 2006; Brown et al., 2016; McLaughlin et al., 2016; Widge et al., 2017). Titrating DBS to be responsive to symptoms as they arise and prospectively develop may be a more effective approach for treating symptoms and reducing side effects of stimulation. This approach is commonly called “closed loop” or “adaptive” DBS and may prove advantageous in the treatment of OCD and many other psychiatric disorders (Barrett, 2017).

Development of an adaptive DBS (aDBS) system would require identification of the dysfunctional brain signals, or “biomarkers,” related to symptoms, an understanding of how electrophysiological biomarkers might shift acutely and chronically, and technology to control stimulation based on detection of relevant biomarkers. Each point poses significant challenges for the field. Psychiatric illnesses are often characterized by multiple behavioral phenotypes even within individuals with the same diagnosis (Ahmari and Dougherty, 2015). This heterogeneity suggests that effective biomarkers will not likely map to overarching diagnoses, but instead to specific behavioral constructs such as the those defined by the Research Domain Criteria Matrix (e.g., reward learning, working memory, attention) (Insel et al., 2010). Further, once electrophysiological biomarkers are identified, it will be important to understand how these signals may change over time in order to develop biomarker detection algorithms that adapt to these changes (Wu et al., 2018). While some existing DBS technology has responsive capabilities (i.e., stimulation in response to biomarker detection), further innovation is required to develop adaptive capabilities (i.e., algorithm adaptation in response to evolving brain states). Additionally, existing technology may require further innovation to provide the recording capabilities, stimulation specificity, battery life, and computational power needed to fully realize aDBS as a viable therapy for neuropsychiatric illness.

In this article we aim to: (1) highlight the potential advantages of a responsive DBS system over existing treatments for OCD and other neuropsychiatric illnesses; (2) propose methods of identifying control signals for responsive neuromodulation; and (3) discuss current and future tools that can be used in the development of responsive neuromodulation systems.

## Adaptive Neuromodulation for Symptoms of Neuropsychiatric Illnesses

### DBS as a Tool for Disrupting Corticostriatal Pathways

To develop an aDBS system for treatment of symptoms of mental disorders, it is important to better understand the circuitry and pathological processing involved, and thus how DBS could intervene. Aberrant activity in corticostriatal loops has been hypothesized to be an underlying etiology for several neuropsychiatric conditions involving reward pathway dysfunction, including OCD, addiction, anxiety, and depression (Greenberg et al., 2003; Milad and Rauch, 2012; Ahmari and Dougherty, 2015; Peters et al., 2016; Sharma et al., 2016). The STN, caudate nucleus (CN), and ventral striatum (VS) are recognized as key players in the corticostriatal network. Specifically, the involvement of STN in inhibitory control and decision threshold regulation has been shown via imaging, electroencephalography (EEG), and intracranial electrophysiology in humans (Aron et al., 2007; Cavanagh et al., 2011; Whelan et al., 2012; Zavala et al., 2014; Frank et al., 2015), primates (Aron et al., 2007; Cavanagh et al., 2011; Whelan et al., 2012; Zavala et al., 2014; Frank et al., 2015) and rodents (Schmidt et al., 2013). Abnormal activity in the CN has been shown in individuals with depression (Price and Drevets, 2012), and the VS plays a critical role in reward processing and prediction as well as reward prediction errors (Pagnoni et al., 2002; Tanaka et al., 2004).

There has been growing interest in clinically targeting these subcortical regions for treatment refractory depression (TRD) and OCD, as well as substance use disorder (SUD) via DBS. In OCD specifically, white matter fibers that connect the VS (Norberg et al., 2008; Lehman et al., 2011) with medial prefrontal cortical regions have been the main target of DBS electrodes. The nucleus accumbens (NAc) is a subpart of the VS and has been used as a DBS target for OCD. In a seminal study of NAc DBS for OCD, DBS was found to normalize NAc activity, and was associated with a reduction of OCD symptoms (Figee et al., 2013). Interestingly, targeting the VS has also been suggested to be effective for treatment of TRD (Malone et al., 2009; Bewernick et al., 2012). In OCD patients, STN DBS has also been productive in providing therapy by downregulating decision thresholds and sensitivity to uncertainty (Voon et al., 2017).

It is perhaps unsurprising that the circuitry thought to underlie TRD, OCD, and SUD is overlapping, as individuals with these disorders often present with overlapping phenotypes and symptoms, especially involving reward dysfunction. Specifically, a defining feature of MDD is a pervasive lack of interest in enjoyable activities, whereas individuals with SUD excessively pursue a substance in a way that is disproportionate to the pleasure derived from it (Baskin-Sommers and Foti, 2015). In OCD, patients often overvalue the impact undesirable circumstances have on potential rewards (e.g., increased delay discounting) (Figee et al., 2011; Baskin-Sommers and Foti, 2015; Voon et al., 2017). Elucidation of the mechanistic overlap across various mental disorders may require a paradigm shift away from thinking of disorders as separate entities. Instead, it may be more productive to consider mental illness in terms of functionality across various domains, including cognitive processes in reward, decision-making, attention, and other goal-directed behaviors (Insel et al., 2010; Widge et al., 2017). This paradigm shift may foster a better understanding of the role that subcortical nodes play in corticostriatal circuitry across specific symptoms common to various mental disorders.

While the VC/VS has historically been the primary DBS target for OCD, future work calls for optimizing surgical targeting through tractography, as has been demonstrated for subcallosal cingulate (SCC) DBS for TRD (Riva-Posse et al., 2018). Additional key corticostriatal regions should be explored as potential DBS targets for OCD, including the anterior cingulate cortex (ACC), orbitofrontal cortex (OFC), and subgenual cingulate (Brodmann area 25) (Kopell and Greenberg, 2008; Haber and Heilbronner, 2013). Each of these regions have been targeted with DBS with varying levels of success for neurological disorders including TRD (Johansen-Berg et al., 2008; Rao et al., 2018) and chronic pain (Boccard et al., 2014; Riva-Posse et al., 2018). DBS applied to alternative corticostriatal nodes has the potential to optimally modulate corticostriatal activity to provide enhanced therapeutic benefits.

### aDBS for Treatment of OCD Symptoms

While DBS holds promise as a future treatment for many neuropsychiatric disorders including TRD and SUD, the only approved neuropsychiatric indication of DBS is OCD, via a humanitarian device exemption. Therefore, to limit the scope of this paper, the current section focuses on the application of aDBS to OCD symptoms.

Within current DBS paradigms, challenges arise from both the occurrence of stimulation-related side effects and the fluctuating nature of OCD symptom severity. In particular, hypomanic symptoms are among the most common side effects experienced by patients receiving DBS for OCD symptoms (Kisely et al., 2014; Alonso et al., 2015), and is often managed by reducing stimulation amplitude (Widge et al., 2016). On the other hand, if stimulation amplitude is set too low, it may provide insufficient relief of OCD symptoms. Stimulation must therefore be carefully tuned to balance the therapeutic benefit of higher stimulation amplitudes against the risk of inducing hypomania, anxiety, or impulsivity (Kisely et al., 2014; Alonso et al., 2015).

The delicate task of tuning stimulation is made more difficult by the slow response times between a change in stimulation settings, the onset of hypomania, recognition and report of the hypomanic state, and an appointment for DBS re-programming. Although this process does represent a form of closed-loop control of DBS where the clinician integrates clinical measures to control stimulation, the timescale of the response may be measured in days, weeks, or longer, and may be disruptive to a patient’s daily life. Furthermore, because OCD symptoms can vary over time and with context (e.g., environmental triggers), there may not be a single ideal stimulation amplitude for each patient. Changes in symptoms, side effects, and symptom severity fluctuate rapidly, and are often not addressed by infrequent clinical visits. While patients themselves could be given limited control of stimulation parameters (e.g., amplitude), due to the delicate nature of the circuitry involved, this could lead to undesirable outcomes.

An adaptive form of DBS could offer improved therapy for OCD by sensing changes in neural activity and adjusting stimulation parameters in response. For example, a system able to detect the onset of stimulation-related hypomania and related impulsivity could react by automatically lowering stimulation amplitude to a more appropriate level. If the aDBS system were also able to detect increases in the severity of OCD symptoms or detect maladaptive cognitive states that would benefit from DBS, stimulation amplitude could be increased in these contexts. While amplitude is perhaps the most well-understood stimulation parameter, future work may uncover different sets of frequencies, patterns, or phase-locking strategies (Cagnan et al., 2017) that are optimal in different contexts. However, even with perfect knowledge of when to stimulate, future work will be necessary to elucidate exactly how to adjust stimulation parameters to optimally relieve symptoms, and how different types of stimulation affect brain network activity.

Unlike the manual tuning process used today, this closed-loop aDBS system would not require intervention from the patient or clinician. An aDBS system would likely respond on faster timescales, limited primarily by the time required to detect the biomarker. Adjusting stimulation on-demand could improve efficacy by providing therapy only when needed and reducing the risk of side effects when therapy is not needed.

## Identifying Control Signals

### Overview

Identification of stable, definitive, neural signatures, or “biomarkers,” of disease-relevant states is an essential component of an aDBS system for neuropsychiatric disorders. A challenge for identifying disease-specific biomarkers is that behavioral symptoms are not homogenous for single diagnoses and overlap between multiple diagnoses (Insel et al., 2010). Additionally, disease states related to neuropsychiatric disorders are not static; symptoms advance and retreat over periods as short as seconds to days. A way over this hurdle may be to identify personalized biomarkers that pertain to transient, maladaptive behavioral states, rather than biomarkers of a static, constant overall disease state (Widge et al., 2017).

Much of what we know about neural signatures of neuropsychiatric disorders has been derived from fMRI paradigms designed to expose differences between healthy and diseased brain states (Greicius, 2008; Insel et al., 2010). This literature has paved the way for identification of relevant circuitry and should guide the search of biomarkers, however, there are caveats to interpretation. In an aDBS system, temporal identification of biomarkers should promptly enable stimulation intervention when maladaptive behaviors are detected. Temporal resolution of fMRI may not be sufficient to capture dynamics of target neural signatures. In addition, fMRI analysis typically involves averaging over trials to reveal significant differences between groups. For the purpose of a responsive aDBS system, neural signatures must be distinctly and reliably separable from that of healthy behaviors at the level of each individual occurrence.

Perhaps unsurprisingly, current closed-loop neural interface platforms rely on electrophysiology (i.e., single-unit activity and local field potential recordings) rather than imaging due to the aforementioned advantages in portability and temporal resolution (Afshar et al., 2013; Sun and Morrell, 2014; Khanna et al., 2015; Ajiboye et al., 2017; Herron et al., 2017). Like imaging, electrophysiological recordings from the brain can be used to uncover patterns of neural activity associated with complex behaviors and/or cognitive states, such as attention, reward evaluation, uncertainty, and conflict resolution. Specifically, patterns of oscillatory activity at single electrodes (e.g., power over a frequency band of interest), and synchrony of activity at single electrodes or groups of electrodes (e.g., coherence, granger causality, and canonical coherence) may encode maladaptive behaviors (Harris and Gordon, 2015). These patterns of activity can be quantified and are often termed as “features” of the neural data. In addition to these univariate signals, biomarkers may also be spatiotemporal clusters of positive and negative activity that covary with precise quantities, such as reward prediction error and uncertainty. Here, data and theory driven methods can be combined in a way that is informed by use of these theoretically meaningful features (Collins and Frank, 2018).

There has been some progress in identification of biomarkers relevant for mental disorders in recent years. For example, beta band coherence between amygdala and hippocampus has been found to predict variation in mood in high anxiety-trait individuals (Kirkby et al., 2018; Sani et al., 2018). In the future, such a biomarker could be leveraged to control DBS during vulnerable mood states across mental disorders.

### Strategy for Uncovering Biomarkers

First, maladaptive behavioral states should be defined and quantified in controlled settings (i.e., psychophysical tasks). Traditionally, clinician administered scales, including the Yale-Brown Obsessive Compulsive Scale (Y-BOCS), have been used to track symptom severity across days or weeks. However, these assessments are too time consuming to be used to quantify behavioral states with adequate time resolution. Therefore, it may be more appropriate to infer behavioral state moment-to-moment from responses during psychophysical tasks, video recordings to enable computer vision-based tracking of expression and pose [e.g., Automated Facial Affect Recognition (AFAR)] (Girard et al., 2015), or physiological measurements, such as heart rate and skin conductance. Task behavior can be used to model various behavioral and cognitive states, including reward evaluation, uncertainty, and error-monitoring.

Next, neural signatures that predict maladaptive behavioral states should be identified. Together, these goals make up the foundation of the field of computational psychiatry (Huys et al., 2016). Identification of neural signatures can be approached in a data driven or theory driven strategy. A data driven approach might involve the development of a machine learning algorithm to parse through a large number of neural features and separate target states from healthy states. A theory driven approach might quantify a dynamic behavioral state of interest and identify meaningful features that correlate with it, or perhaps encode it. Various examples using computational psychiatry show that one can better decode brain state when using parameters derived from mathematical models fit to neural and behavioral data, compared to the same classifier applied to raw data without models (Wiecki et al., 2015).

While psychophysical tasks are a useful tool for studying well-defined behavioral states, it is important that the same processes required for the tasks translate to real world functioning. Translation will likely require comparison of behavior and neural activity in less controlled, more natural settings.

### Error Monitoring: A Promising Control Signal for OCD

This section focuses on biomarker identification for one specific type of abnormal processing that may lead to maladaptive behaviors related to OCD: error-monitoring. We limit the scope to error-monitoring because it is well studied and better understood than other maladaptive behaviors related to neuropsychiatric disorders. Error monitoring involves identifying the difference between a given response and an intended response (i.e., an error). Abnormal error-monitoring processes could explain the pathological doubt and feelings of incompleteness that OCD patients often experience (Pitman, 1987), and there is preliminary supporting evidence showing that biomarkers of error-monitoring may provide information about the current severity of OCD symptoms. A classic electrophysiological marker of error-monitoring is the error-related negativity (ERN), a cortical potential observed in frontal EEG following the commission of an error, or more generally, in response to cognitive conflict. It is well established that the ERN tends to be exaggerated in patients with OCD (Gehring et al., 2000; Endrass and Ullsperger, 2014).

Another biomarker of error-monitoring, in addition to the ERN, is an increase in theta-band power in mediofrontal cortex and NAc that occurs after an error (Cohen et al., 2009; Cavanagh et al., 2010; Lega et al., 2011; Munneke et al., 2015). Figee et al. (2013) have observed a similar increase in theta-band power in frontal EEG during provocation of OCD symptoms. Furthermore, DBS targeting the NAc was found to attenuate the increase in theta band power. This DBS-induced attenuation suggests that shifts in theta-band power may be linked to the severity of OCD symptoms and the therapeutic effects of DBS, making theta-band power a promising control signal for aDBS.

While these findings are promising, to our knowledge, there has been no previous demonstration of an algorithm that dynamically detects failures in error-monitoring with time resolution based on neural recordings; this could be a interesting direction for future research.

### Control Signal Requirements

Requirements for acceptable levels of sensitivity, specificity, and latency for biomarker detection should depend on clinical application. For example, brain states associated with certainty and conflict during decision making are transient and may require immediate action timescales of less than one second. Brain states associated with provoked anxiety might wax and wane over minutes (e.g., anxiety, post traumatic stress disorder, OCD), while brain states associated with depression or mania might evolve over days (e.g., bipolar disorder, TRD). In addition to achieving accurate detection, closed-loop decoding algorithms must be reliable over time, which may require frequent recalibrations and online updating to reduce nonstationarities (Dangi et al., 2013; Jarosiewicz et al., 2015). For example, shifts in electrophysiological recordings over time may require onboard biomarker detection algorithms to adapt by automatically adjusting feature weights in response. Algorithm adaptation capabilities will enable aDBS in its true form, and may present opportunities to treat neurological disorders for which DBS or responsive DBS has previously been unsuccessful.

## Opportunities for Designing Adaptive Neuromodulation

Neural recordings are crucial for both the development and implementation of an adaptive DBS system. Identifying biomarkers of disease-relevant contexts requires gathering sufficient amounts of high quality neural data from the brain, extracting useful features from neural activity, and comparing neural features with ground-truth knowledge of the current context. Because proposed mechanisms and proposed biomarkers of neuropsychiatric illness frequently involve interactions between deep brain structures and cortical areas, simultaneous recording from both of these areas is ideal. A practical adaptive DBS system must be able to chronically record from one or more regions and use the recorded data to decide how and when to stimulate.

There are a number of available approaches for recording such data and identifying biomarkers during development of an aDBS system, each with benefits and tradeoffs.

### EEG

Electroencephalography recording is a non-invasive technique that is widely used for large groups of controls and participants with a variety of neurological disorders. EEG can be used to collect hours of data in a laboratory setting, and can be repeated at various time points in a longitudinal study. Unfortunately, EEG is impractical for chronic or ambulatory use, and therefore could not be used to control an aDBS system. Although EEG data has lower spatial and temporal resolution than intracranial neural recordings, it can be used to identify preliminary biomarkers that should later be further investigated through other methods. For example, there is evidence that frontal midline theta observed via EEG reflects anxiety and cognitive control (Cavanagh and Shackman, 2015), as well as decisional conflict (Frank et al., 2015).

### Intraoperative and Postoperative

Data can be recorded for short periods of time during a DBS lead implantation procedure from (1) the contacts of the DBS lead itself, (2) a depth microelectrode inserted prior to the lead for targeting purposes (Lega et al., 2011), or (3) from ECoG electrodes temporarily placed on the surface of cortex. If the DBS lead is implanted prior to the implantable pulse generator in a separate surgical procedure, there may be an opportunity to record deep brain LFP by connecting the externalized lead to a standard data acquisition system in the days following the first surgery. Compared to intraoperative sessions, this allows for hours of recording time instead of minutes, and allows for more flexibility in the experimental tasks the patient is able to perform and additional forms of data that can be acquired. For example, scalp EEG can be recorded simultaneously and used to detect changes in synchrony and communication between cortical areas and the stimulation site (Cohen et al., 2009; Horschig et al., 2015).

These intraoperative and immediately postoperative recording sessions both provide opportunities to record data from the same neuropsychiatric patient population that would use the aDBS system, as well from control patients receiving DBS for other indications. These sessions also create a platform for briefly testing stimulation paradigms and closed-loop control algorithms.

### Pre-surgical Monitoring

Patients awaiting resection surgery for intractable epilepsy provide a valuable source of high quality ECoG and/or depth LFP recordings, recorded continuously for days or weeks at a time, including during behavioral tasks. Because the patient population is epileptic, neuropsychiatric disorders can only be studied by chance, if comorbid with intractable epilepsy. Non-etheless, biomarkers relevant for neuropsychiatric indications related to variation in mood have been identified using this datasource (Kirkby et al., 2018). In the future, similar in-patient electrophysiological monitoring prior to or at the same time as DBS surgery could be an valuable opportunity to identify biomarkers and optimize DBS parameters before chronic use.

### Opportunities for Chronic Recording and Stimulation With Implanted Devices

Deep Brain Stimulation implants that can record as well stimulate are not only essential for the implementation of aDBS systems, but also highly valuable for identifying biomarkers and prototyping aDBS. Currently, there are two existing hardware platforms that are capable of responsive neuromodulation: the NeuroPace RNS (Mountain View, CA, United States), and the Activa PC+S (Medtronic, Minneapolis, MN, United States).

The NeuroPace RNS is a fully implanted, closed-loop, FDA approved device that reduces epileptic seizures by applying electrical stimulation based on detection of seizure onset (Sun and Morrell, 2014). The NeuroPace RNS includes two separate implantable stimulation and sensing leads, and is configured by default for closed-loop control. Additionally, work by Wu et al. (2018) has demonstrated feasibility of using RNS to suppress binge eating in mice by delivering neurostimulation based on detection of NAc delta oscillations related to reward anticipation (Kirkby et al., 2018). This preclinical work has opened the door for future clinical work using RNS to downregulate pathological impulsivity in humans.

The Medtronic Activa PC+S is an FDA approved device that offers the same therapeutic capabilities as the more commonly used Activa PC, with the added capability of recording through the lead or through additional chronically implanted ECoG electrodes (Stanslaski et al., 2012). The Activa PC+S enables a “computer-in-the-loop” approach to prototyping aDBS in the clinic by wirelessly streaming neural data to a computer as it is recorded (Rosin et al., 2011; Little et al., 2013; Herron et al., 2017). In this approach, an external computer receives and processes the data, applies a biomarker detection algorithm, and wirelessly transmits stimulation commands back to the implant. The use of a powerful external computer allows for a great degree of flexibility in choice of algorithms, and makes it possible to use external data sources, like EEG or behavioral task performance, to affect stimulation.

Later stage prototypes and final implementations of aDBS should transition from a computer-in-the-loop design to a fully implanted system, where all recording, data processing, biomarker detection, and control decisions are performed on hardware within the body (Khanna et al., 2015). This avoids the increased power consumption and latency caused by wireless communication with external devices. This is already developed, in part, in the form of the NeuroPace RNS. However, true aDBS will require additional hardware improvements and online adaptation algorithms. Both the Activa PC+S and NeuroPace are primary cell systems and do not allow data streaming for long periods of time. Ideally, the device should be rechargeable to extend the lifetime of the device, and should be capable of data streaming to aid in algorithm adjustment when necessary.

## Conclusion

Development of a neuromodulation system that operates by responding to anticipated or detected abnormal neural activity may be a way forward for improving treatment efficacy of mental illness. DBS has shown promise for treating mental illness over the last two decades, however, results of trials have been mixed. Patient heterogeneity, failed surgical targeting, lack of stimulation specificity, and the open-loop nature of the current DBS paradigm may all in part explain these mixed results. Adaptive stimulation has the potential to address these issues. In an adaptive system, stimulation parameters would be adjusted based on the current state of the patient, and state detection algorithms would adapt over time. In this way, stimulation would be provided only when needed, which might prevent harmful side-effects caused by stimulating at the wrong times.

Dysfunction in the corticostriatal loop is implicated across various mental disorders, and DBS studies for mental disorders commonly target regions in this network. For example, as we have discussed, pathological activity in the VC/VS and STN is implicated for OCD, TRD, and SUD, and DBS targeting these regions has been shown to improve symptoms. It’s not surprising that the underlying circuitry is overlapping, as different diagnoses often present with overlapping phenotypes. Thinking about mental illness not in terms of symptoms, but instead in terms of functionality across various domains, including reward evaluation, emotion regulation, decision making, and attention, may help to elucidate the underlying, overlapping mechanisms of various mental disorders.

We recommend approaching the search for biomarkers using this mindset by searching for biomarkers of specific maladaptive behaviors that affect functionality in everyday life. Psychophysical tasks are well-defined, controlled ways to quantify maladaptive behaviors. Recording from the brain during psychophysical tasks presents opportunities to identify neural signatures of the mental processes required to perform the task. Failures to perform the task may indicate maladaptive behaviors that affect a patient’s ability to function in the real world. An ideal biomarker will sensitively, specifically, and reliably detect maladaptive behaviors at every occurence, so stimulation intervention can be applied to augment function as needed.

There are many existing opportunities for neural recording and stimulation that will inform both identification of biomarkers and development of aDBS technology. Opportunities with EEG, intraoperative and postoperative recording, and presurgical monitoring should be harnessed to augment data from more “case-relevant,” chronic recordings from implantable hardware. Additionally, computer-in-the-loop configurations can provide greater computing power and flexibility than existing implantable hardware for prototyping closed-loop algorithms. We believe that a system that can record simultaneously from subcortical and cortical structures is ideal, as such a device could sense the changes in communication in corticostriatal loops implicated in neuropsychiatric disorders. If we cannot record simultaneously from cortical and subcortical structures, a single device implanted subcortically should target other regions of interest through passing fiber tracts defined by tractography, imaging, or electrophysiological mapping.

We have detailed how an aDBS system might function in the treatment of OCD, as we believe aDBS for OCD is the closest to fruition out of the neuropsychiatric disorders discussed here. However, as the mechanisms underlying each diagnosis are unveiled through further research, we think that aDBS has the potential to make an impact in treatment of mental disorders and other domains.

## Author Contributions

NP, ERM, AA, and AB-A prepared the manuscript with the guidance of DB. SS, AV, EM, ES, MF, NM, JC, and WG provided intellectual contributions through discussions and feedback.

## Conflict of Interest Statement

Medtronic donates devices for an NIH Funded Research study (WG). MF is a consultant for Hoffman La Roche Pharmaceuticals. The remaining authors declare that the research was conducted in the absence of any commercial or financial relationships that could be construed as a potential conflict of interest.
